# A novel prognostic model for hepatocellular carcinoma based on pyruvate metabolism-related genes

**DOI:** 10.1038/s41598-023-37000-8

**Published:** 2023-06-16

**Authors:** Qingmiao Shi, Chen Xue, Yifan Zeng, Xinyu Gu, Jinzhi Wang, Lanjuan Li

**Affiliations:** State Key Laboratory for Diagnosis and Treatment of Infectious Diseases, National Clinical Research Center for Infectious Diseases, National Medical Center for Infectious Diseases, Collaborative Innovation Center for Diagnosis and Treatment of Infectious Diseases, The First Affiliated Hospital, Zhejiang University School of Medicine, 79 Qingchun Rd., Hangzhou City, 310003 China

**Keywords:** Tumour biomarkers, Cancer, Biomarkers, Diseases, Oncology, Risk factors

## Abstract

Hepatocellular carcinoma (HCC) is the most prevalent form of primary liver cancer, accounting for over 90% of cases. As pyruvate metabolic pathways are often dysregulated in cancer cells, investigating pyruvate metabolism-related genes may help identify prognostic gene signature and develop potential strategies for the management of patients with HCC. The mRNA expression profile, gene mutation data, and clinical information of HCC were obtained from open-source databases. A list of pyruvate metabolism-related genes was downloaded from the MSigDB dataset. Our findings revealed that certain pyruvate metabolism-related genes had copy number variations and single nucleotide variations in patients with liver cancer. Based on pyruvate metabolism-related genes, we stratified patients with HCC into three subtypes with different prognoses, clinical features, mutation profiles, functional annotation, and immune infiltration status. Next, we identified 13 key pyruvate metabolism-related genes significantly correlated with the prognosis of HCC using six machine learning algorithms and constructed a risk model. We also observed that the risk score was positively associated with a worse prognosis and increased immune infiltration. In summary, our study established a prognostic risk model for HCC based on pyruvate metabolism-related genes, which may contribute to the identification of potential prognostic targets and the development of new clinical management strategies for HCC.

## Introduction

Primary liver cancer encompasses hepatocellular carcinoma (HCC) and intrahepatic cholangiocarcinoma, with HCC being the most prevalent type, accounting for over 90% of cases^1,2^. The pathogenesis of HCC is a multifactorial, multistep process influenced by environmental and dietary factors, such as hepatitis B and hepatitis C virus infections, excessive alcohol consumption, and metabolic diseases^3^. Traditional treatment methods and emerging therapies have diversified liver cancer treatment options, including surgery, radiotherapy, chemotherapy, intervention, targeted drugs, immunotherapy, and other approaches^4^. Despite advances in treatment, the HCC recurrence rate remains high even in patients with HCC who have undergone liver transplantation^5^. As one of the lowest-survival cancers in the world, liver cancer is predicted to cause 1.3 million deaths in 2040, compared with the statistics of 830,200 in 2020^6^. Given the high recurrence and mortality rates, establishing a new model predicting the HCC prognostic risk is critical.

Pyruvate plays a crucial role in biochemical metabolism, particularly in eukaryotic glycolysis^7^. As the end-product of glycolysis in humans, pyruvate can be reduced to lactic acid in the cytoplasm to generate energy or oxidized to carbon dioxide, water, and energy via the tricarboxylic acid cycle in the mitochondria^8^. Pyruvate also contributes to the conversion of sugar, fat and amino acids in the body through the acetyl-CoA and tricarboxylic acid cycle, making it a pivotal element in the metabolic relationship of the three major nutrients. Any genetic mutation that occurs during pyruvate metabolism may lead to various diseases. For instance, *HIF1,* the regulator of pyruvate metabolism, and the cell regulator *p53* can promote the Warburg effect by regulating abnormal pyruvate metabolism, thereby playing a significant role in the formation and progression of cancer^9–11^. Additionally, pyruvate metabolic abnormalities are observed in chronic progressive diseases, such as neurodegenerative diseases, heart failure, and diabetes^8^.

Indeed, energy metabolism reprogramming has been shown to serve as one of the hallmarks of cancer^12^. Aerobic glycolysis, also known as the Warburg effect, was first reported in HCC in the 1920s, which refers to the conversion of glucose to lactic acid in tumor cells even in the presence of sufficient oxygen. This phenomenon differs from normal cells in which oxidative phosphorylation catabolizes glucose^13^. The regulation of pyruvate metabolism is the decisive point in determining whether mitochondrial carbohydrate oxidation occurs or aerobic glycolysis (Warburg effect)^10^. Several rate-limiting enzymes in the glycolysis pathway, such as hexokinase 2, phosphofructokinase 1, and pyruvate kinase M2 (PKM2), play essential roles in the regulation of aerobic glycolysis in HCC and respond to changes in signaling pathways such as c-Myc, HIF-1α, and PI3K/Akt^14,15^. Qiuran Xu and her colleagues demonstrated that HSP90 could promote glycolysis and proliferation of HCC cells while inhibiting apoptosis by binding to PKM2, thereby promoting HCC growth^16^. Abnormal pyruvate metabolism has also been linked to the maintenance of tumor microenvironment, immune evasion of tumor cells, and hypoxia-inducible factor (HIF)-mediated metabolic reprogramming is involved in drug resistance in HCC^17,18^. As pyruvate synthesis and consumption are differentially regulated in cancer cells, a systematic analysis of pyruvate metabolic pathways may help identify prognostic gene signature and develop potential strategies for the early diagnosis and management of patients with HCC.

In this study, we determined three molecular subtypes of patients with HCC using consensus clustering analysis based on pyruvate metabolism-related genes. Then the differentially expressed genes (DEGs) were screened between the three subtypes. We established a prognostic evaluation method for HCC using multivariate Cox analysis based on pyruvate metabolism-related genes. We further evaluated the differences between different risk groups in clinicopathological features, signaling pathways, immune scores, and RiskScores at the single-cell level.

## Results

### Mutation analysis and gene expression of pyruvate metabolism-related genes in HCC

**Figure 1:**
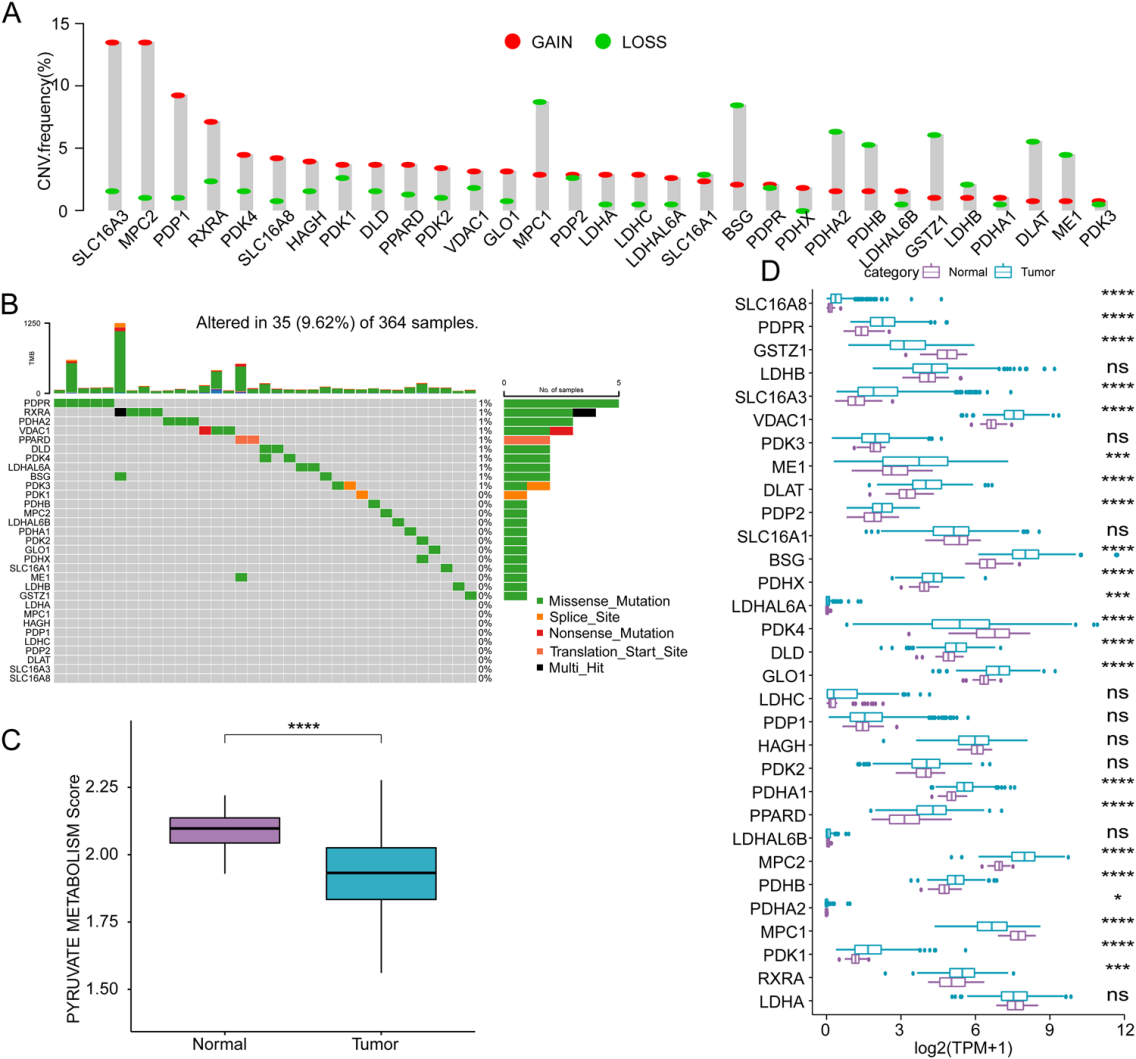
Mutation analysis and gene expression of pyruvate metabolism-related genes in patients with HCC from TCGA-LIHC cohort. (**A**) The frequency of CNV mutations in genes related to pyruvate metabolism. (**B**) The proportion of SNV mutations in genes related to pyruvate metabolism. (**C**) Comparison of pyruvate metabolic pathway score between tumor tissues and adjacent nontumor tissues. (**D**) The expression level of pyruvate metabolism-related genes in tumor tissues and adjacent nontumor tissues.

To examine the involvement of the pyruvate metabolic pathway in HCC, we analyzed the gene copy number variations (CNV) mutation data of HCC from the Cancer Genome Atlas (TCGA) database. Our analysis revealed that several genes, such as *SLC16A3*, *MPC2*, *PDP1*, and *RXRA*, had a high proportion of amplification. In contrast, others, such as MPC1, BSG, PDHA2, PDHB, GSTZ1, DLAT, and ME1, had a high proportion of censoring (Fig. 1A). The waterfall diagram revealed that 35 (9.62%) of 364 samples exhibited mutations in pyruvate metabolism-related genes (Fig. 1B). Missense mutations were the most common type of mutations detected in the pyruvate metabolism-related genes. In contrast, translation start site mutation, splice site mutation, multi-hit, and nonsense mutation were only observed in individual samples. Next, we calculated the pyruvate metabolic pathway score using the the single sample gene set enrichment analysis (ssGSEA) method and found that the tumor tissues exhibited lower score than adjacent tissues (Fig. 1C). Additionally, we compared the expression levels of genes involved in the pyruvate metabolic pathway and found significant differences in most of the genes between HCC and adjacent tissues (Fig. 1D).

### Construction of molecular subtypes based on pyruvate metabolism-related genes

**Figure 2:**
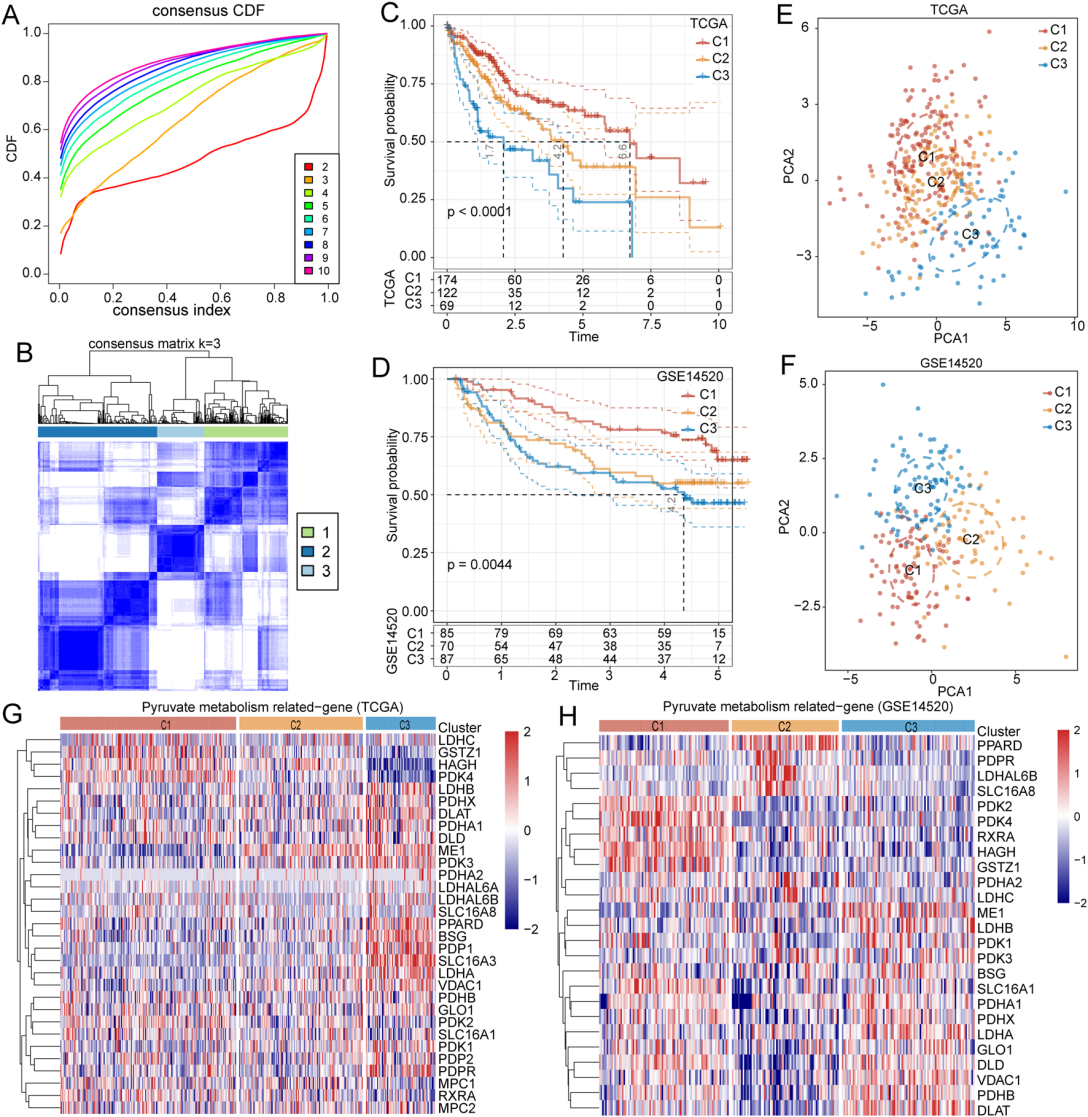
Establishment of molecular subtypes of HCC based on pyruvate metabolism-related genes. (**A**) The curve of the cumulative distribution function. (**B**) Clustering heatmap of samples when consensus matrix k was 3. (**C**-**D**) Kaplan–Meier survival analysis of the three clusters in the TCGA (**C**) and GSE14520 (**D**) datasets. (**E**–**F**) Principal component analysis of the three clusters in the TCGA (**E**) and GSE14520 (**F**) datasets. (**G**-**H**) Expression heatmap of pyruvate metabolism-related genes in the TCGA (**G**) and GSE14520 (**H**) datasets.

**Figure 3:**
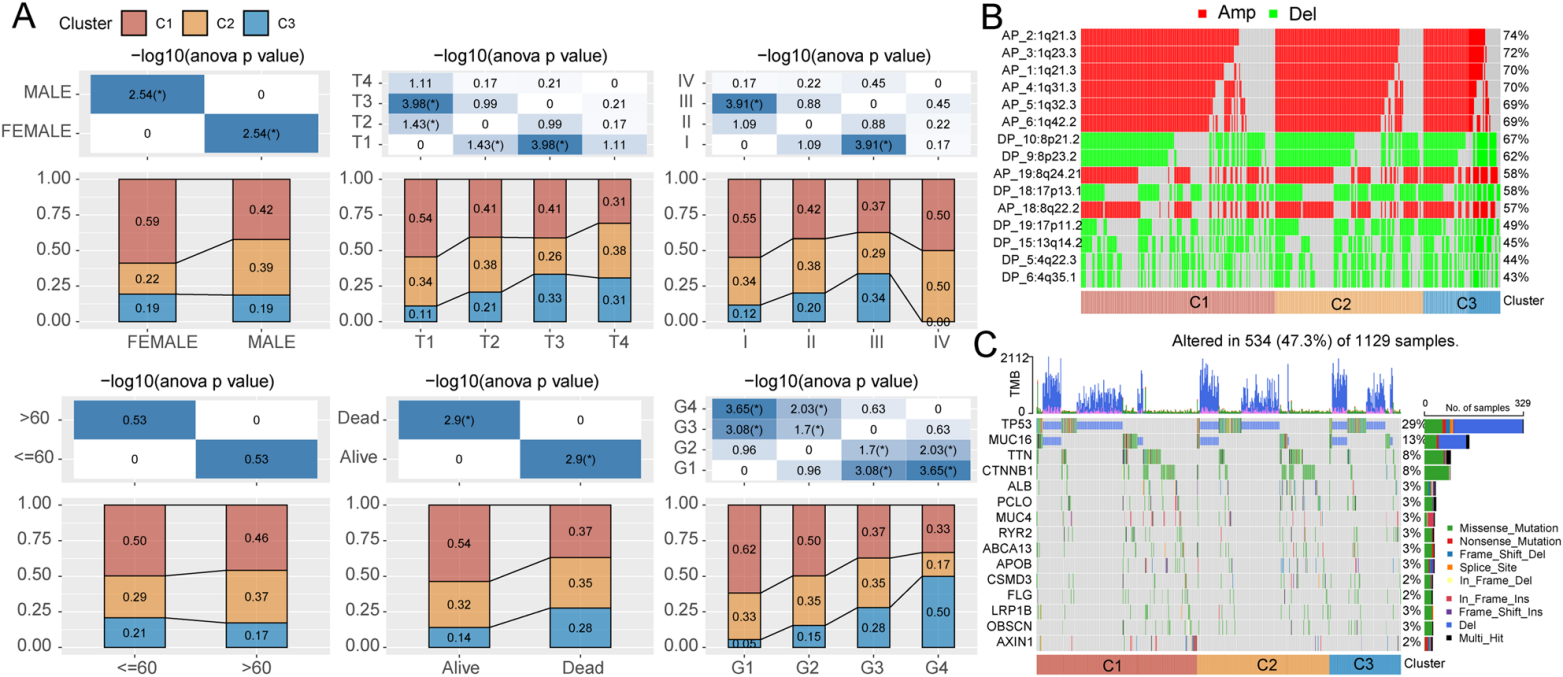
Clinical features and mutation characteristics of three molecular clusters. (**A**) Comparison of clinical and pathological features in the three clusters, including gender, T stage, TNM stage, age, outcome, and pathological grade. (**B**) Top 15 sites of CNV mutation (amplification and deletion) in the three clusters. (**C**) Top 15 genes with SNV and CNV mutations in the three clusters.

Next, we performed consensus clustering of genes related to pyruvate metabolism, with the optimal number of clusters determined based on cumulative distribution function (CDF) analysis. The CDF Delta area curve indicated that the clustering result was more stable when the cluster was selected as 3 (Fig. 2A). The clustering heatmap of the three clusters is shown in Fig. 2B. Three-dimensional principal component analysis suggestes a conspicuous discrimination between the three molecular subtypes (Fig. S1). Kaplan–Meier survival analysis revealed a significant correlation between the three subtypes and the prognosis of patients with HCC, with C3 subtype exhibiting the worst prognosis (Fig. 2C). These findings were verified in an independent dataset GSE14520 (Fig. 2D). Principal component analysis analysis suggested that samples of the three subtypes in TCGA (Fig. 2E) and GSE14520 (Fig. 2F) datasets could be distinguished. Besides, the heatmaps showed the expression level of pyruvate metabolism-related genes between the three subtypes (Fig. 2G-H). Next, we explored the clinical features and mutation characteristics of the three subtypes. Results revealed that the C3 subtype had a higher T stage, grade stage, and death rate (Fig. 3A). Furthermore, the heatmap presented the top 15 CNV mutation sites of the three subtypes based on the TCGA database (Fig. 3B), and the waterfall plot illustrated the single nucleotide variations (SNV) and CNV mutations of the top 15 genes (Fig. 3C).

### Potential functional annotation and Immune States of different molecular subtypes

**Figure 4:**
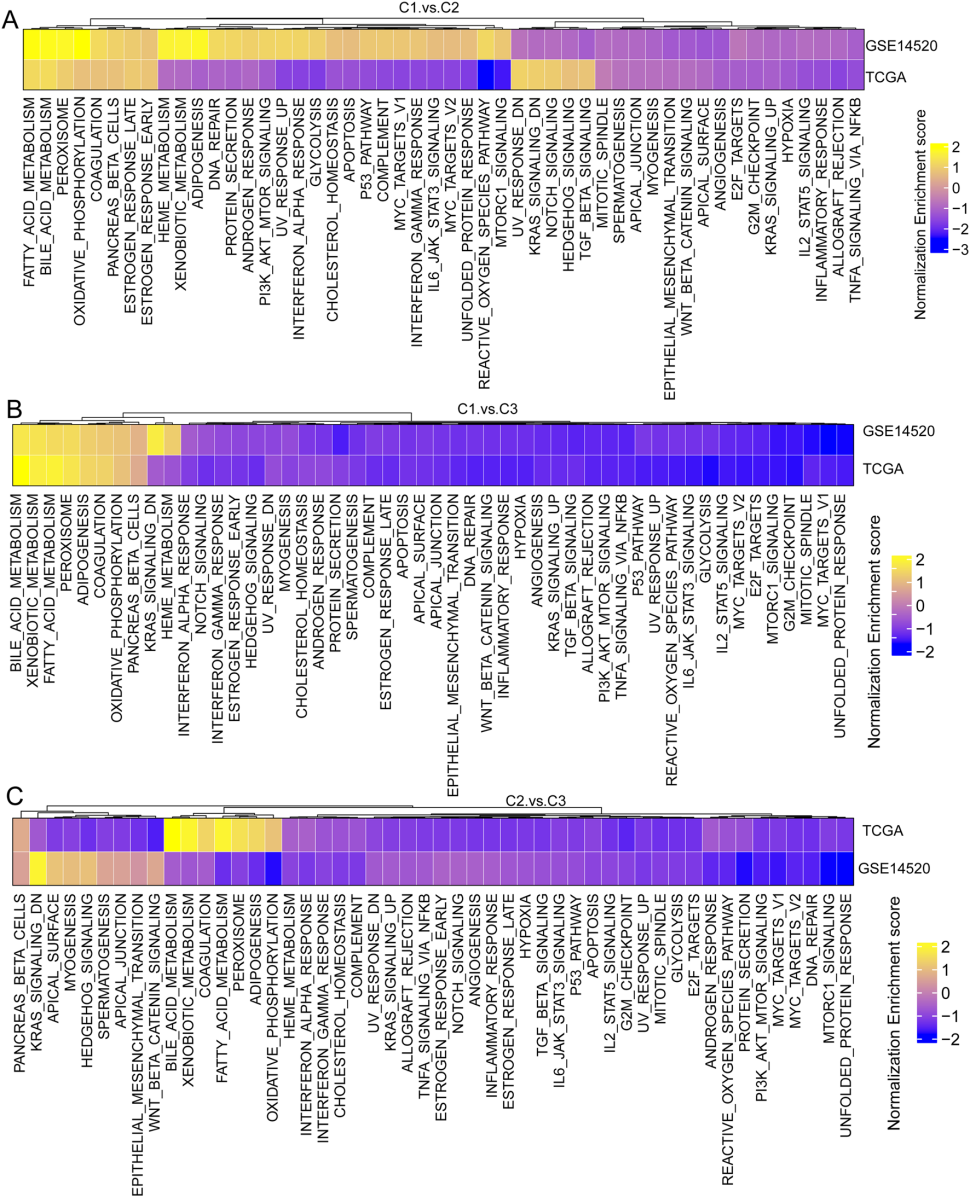
Potential functional annotations of three molecular clusters. (**A**-**C**) Heatmap of enrichment analysis scores of C1 and C2 clusters (**A**), C1 and C3 clusters (**B**), and C2 and C3 clusters (**C**) in the TCGA and GSE14520 datasets.

To investigate the function of different molecular subtypes, we performed enrichment analysis using the HALLMARK gene set in the TCGA and GSE14520 datasets through the GSEA method. The enrichment analysis revealed that each subtype was consistent in both datasets (Fig. 4A-C). All three subtypes were enriched in pathways such as oxidative phosphorylation, bile acid metabolism, and fatty acid metabolism. Moreover, the Kyoto Encyclopedia of Genes and Genomes (KEGG) enrichment analysis based on the TCGA dataset demonstrated that the p53, MAPK, and TGF-β signaling pathways were significantly enriched in the C3 subtype (Fig. S2).

**Figure 5:**
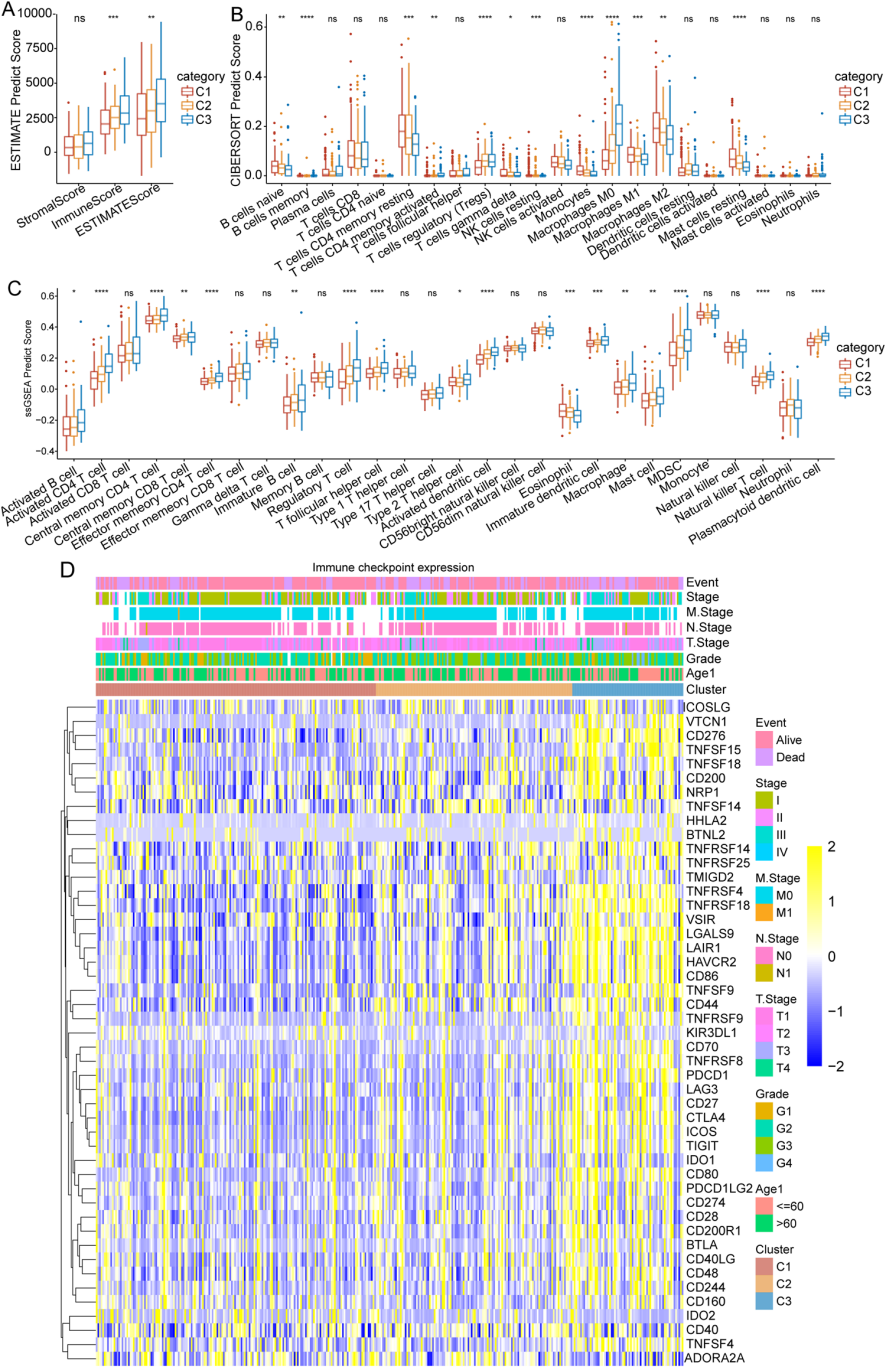
Evaluation of the immune status of the three molecular clusters based on the TCGA dataset. (**A**) Comparison of immune scores predicted by ESTIMATE between the three clusters. (**B**) Comparison of 22 immune cell scores evaluated by CIBERSORT between the three clusters. (**C**) Comparison of 28 immune cell scores assessed by ssGSEA between the three clusters. (**D**) Comparison of immune checkpoint gene expression among the three clusters.

To investigate the immune characteristics, we calculated the immune and stromal scores of the TCGA dataset using the Estimation of STromal and Immune cells in MAlignant Tumour tissues using Expression data (ESTIMATE) method. The results revealed that the C3 subtype, which had the worst prognosis, had the highest immune score (Fig. 5A). Besides, we also used the Cell-type Identification By Estimating Relative Subsets Of RNA Transcripts (CIBERSORT) algorithm to evaluate the scores of 22 immune cells and found significant differences among the three subtypes in some immune cell scores, such as B cells naïve, Monocytes, Macrophages M0-M2, and T cells CD4 memory activated (Fig. 5B). Moreover, the ssGSEA method was used to evaluate the scores of 28 immune cells previously identified in a study^19^. The results showed that the scores of central memory CD4 T cell, T follicular helper cell, and immature B cell were significantly increased in the C3 subtype (Fig. 5C). Finally, we investigated the expression levels of immune checkpoint genes from a previous study^20^, and found that the expression of immune checkpoint genes in the C3 subtype was especially higher than that in C1 and C2 subtypes (Fig. 5D).

### Constructing a risk model based on key genes screened by multiple machine learning algorithms

**Figure 6:**
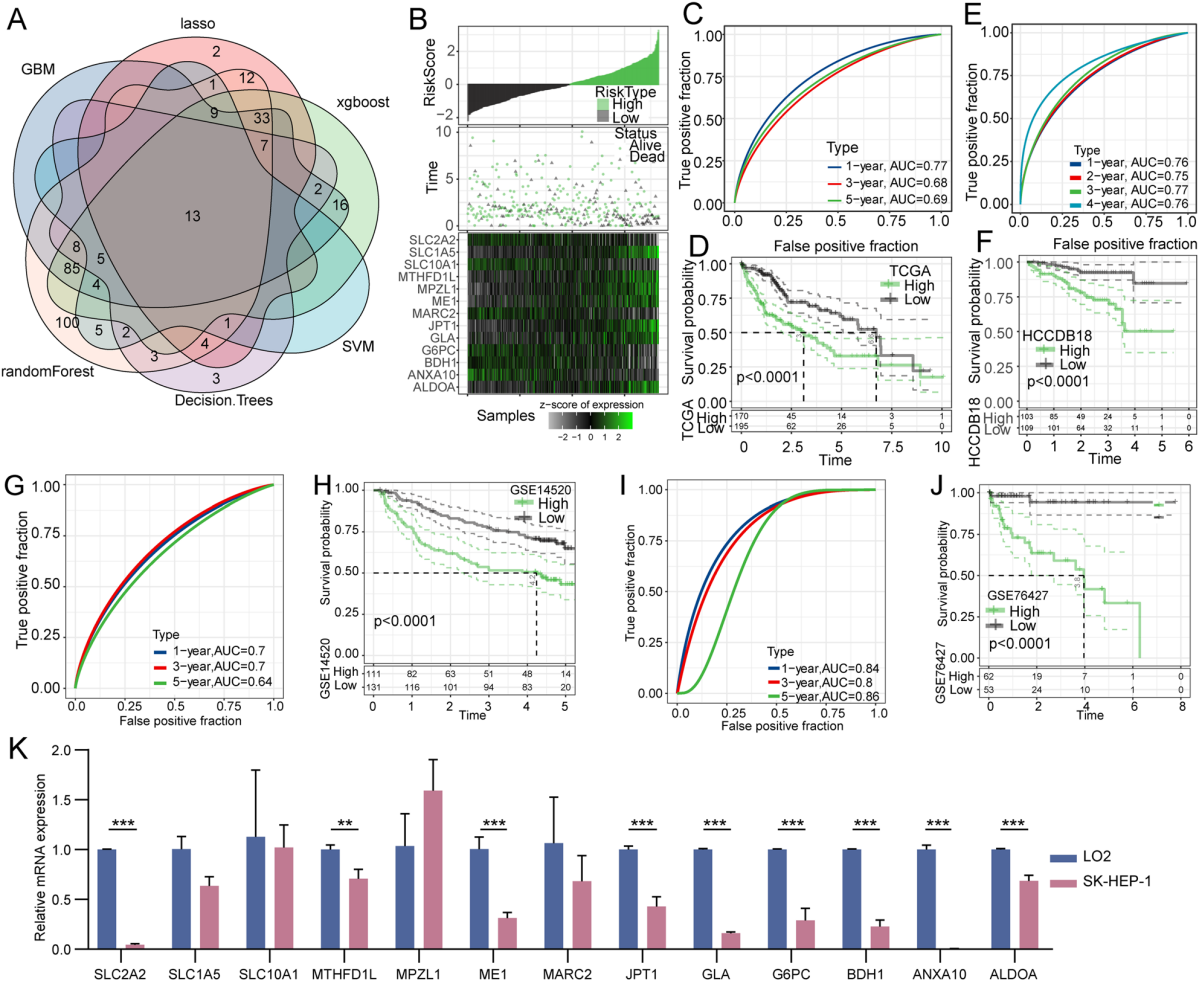
Screening key genes using machine learning algorithms and establishing a risk model. (**A**) Venn diagrams of six machine learning algorithms for screening key genes. (**B**) The distribution of survival status of patients with HCC and the expression of 13 prognostic genes. (**C**) The 1-, 3-, and 5-year AUC curves of the risk model, and (**D**) the Kaplan–Meier survival curves of the high-risk and low-risk groups based on the TCGA dataset. (**E**) The 1-, 2-, 3-, and 4- AUC curves of the risk model and (**F**) the Kaplan–Meier survival curves of the high-risk and low-risk groups based on the HCCDB18 dataset. (**G**) The 1-, 3-, and 5- AUC curves of the risk model and (**H**) the Kaplan–Meier survival curves of the high-risk and low-risk groups based on the GSE14520 dataset. (**I**) The 1-, 3-, and 5- AUC curves of the risk model and (**J**) the Kaplan–Meier survival curves of the high-risk and low-risk groups based on the GSE76427 dataset. (**K**) The expression level of 13 prognostic genes in the SK-HEP-1 and LO2 cell lines by qRT-PCR.

To further identify the key gene sets, the limma package was used to analyze the DEGs between the three subtypes in the TCGA dataset, identifying 1697 DEGs. From this, 390 prognosis-related genes were identified using univariate Cox analysis with a *P*-value threshold of < 0.001. Furthermore, we employed six machine learning methods to screen the gene sets that contributed to the three subtypes, including LASSO, GBM, randomForest, Decision Trees, SVM, and xgboost, which identified 13 genes using overlapping analysis (Fig. 6A). Moreover, we used TCGA cohort as the training set and calculated the risk score of each sample based on the expression level of these 13 genes (Fig. 6B). Receiver operator characteristic (ROC) analysis demonstrated that a good predictive effect of the risk score on the prognosis of 1-, 3-, and 5-year survival (Fig. 6C).

The samples were classified as high-risk and low-risk groups based on the Zscore value of the risk score. Kaplan–Meier survival analysis exhibited a significant difference between the survival of the two groups (Fig. 6D). To validate the robustness of the risk model, we tested it on HCCDB18, GSE14520, and GSE76427 datasets. The ROC curve and K-M analysis were performed based on the HCCDB18 (Fig. 6E-F), GSE14520 (Fig. 6G-H), GSE76427 (Fig. 6I-J) datasets, and the results indicated that the model had good stability. To explore the mRNA expression level of the 13 genes, we performed qRT-PCR on LO2 and SK-HEP-1 cells, revealing significant downregulation of most genes in SK-HEP-1 cells compared to LO2 cells, including *SLCA2*, *MTHFD1L*, *ME1*, *JPT1*, *GLA*, *G6PC*, *BDH1*, *ANXA10*, and *ALDOA* (Fig. 6K).

### The correlation between risk model and clinical features

**Figure 7:**
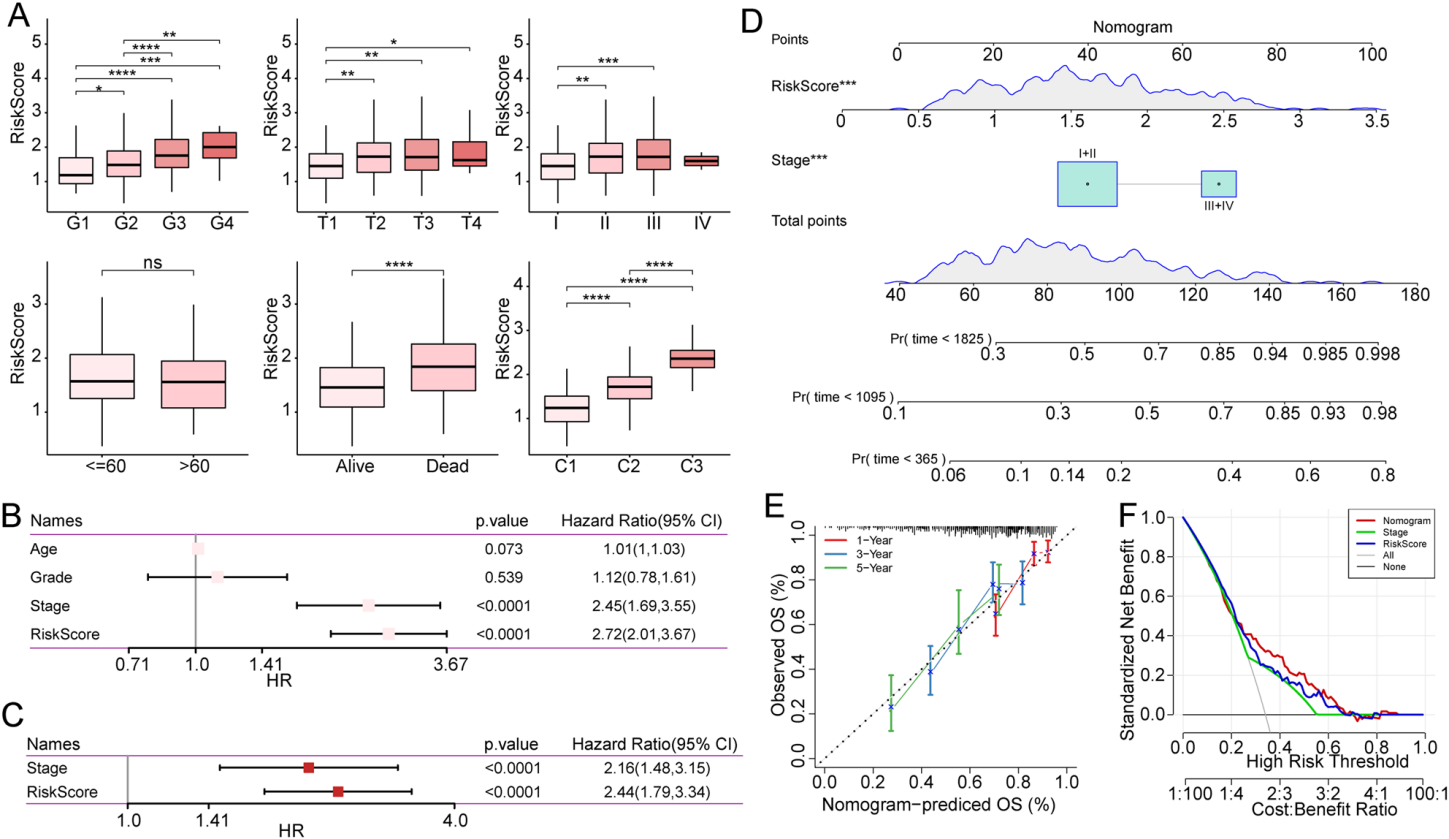
Association between risk score and clinical characteristics. (**A**) Evaluation of risk score of different clinical features. (**B**) Univariate Cox analysis of risk score and clinicopathological characteristics. (**C**) Multivariate Cox analysis of risk score and Stage. (**D**) Nomogram model. (**E**) The 1-, 3-, and 5-year calibration curves of the Nomogram. (**F**) Prediction efficiency of Nomogram, Stage, and Riskscore.

To investigate the association between the risk model and clinical characteristics, we first compared the risk scores of different clinical features using the Wilcoxon test. We found that as the clinical grade increased, the risk score also increased (Fig. 7A). Both univariate (Fig. 7B) and multivariate Cox analyses (Fig. 7C) suggested that Stage and risk score were independent prognostic factors. To evaluate the accuracy of the risk model, we generated a calibration curve, which demonstrated good prediction performance of the Nomogram (Fig. 7D). Moreover, the decision curve of the Nomogram was adopted to validate the reliability of the model (Fig. 7E). Finally, the standardized benefit analysis uncovered that the nomogram and risk score had powerful survival prediction ability (Fig. 7F).

### Metabolic and immune characteristics under different risk states

**Figure 8:**
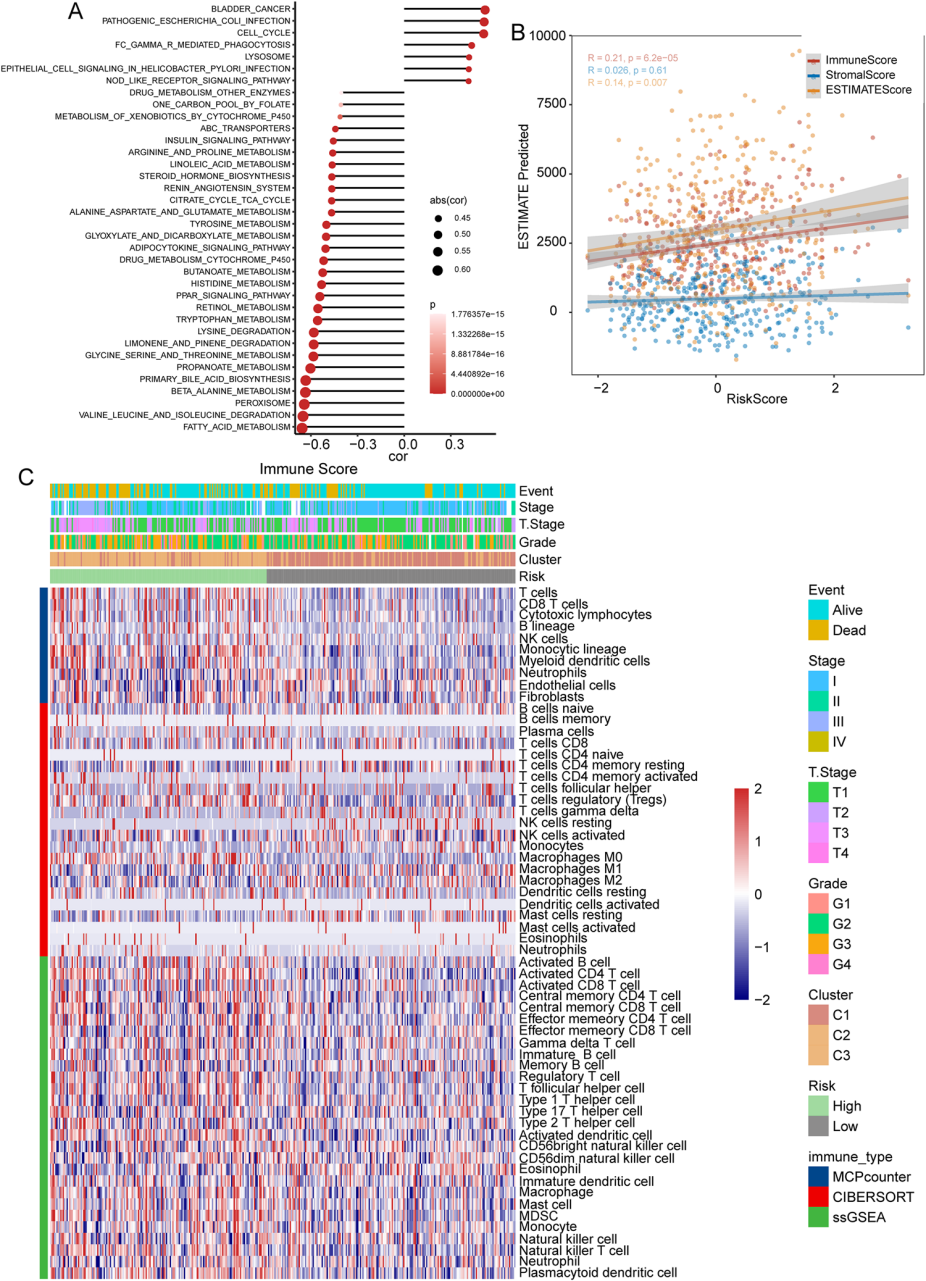
Body characteristics under different risk states in the TCGA database. (**A**) Correlation analysis between pathway score and risk score. (**B**) Correlation analysis between risk score and ESTIMATE predicted immune score. (**C**) Heatmap of immune cell abundance evaluated by various algorithms in the high-risk and low-risk groups.

To examine the metabolic and immune characteristics in different risk states, we explored the correlation between KEGG pathway scores and risk scores. Our findings revealed that various metabolic pathways, such as fatty acid metabolism, β-alanine metabolism, and primary bile acid biosynthesis, were affected to a different extent in the body (Fig. 8A). Next, we assessed the immune score using the ESTIMATE algorithm and found a significant positive correlation between risk score and immune infiltration based on spearman correlation analysis (Fig. 8B). Furthermore, we used MCPcounter, CIBERSORT, and ssGSEA algorithms to evaluate the scores of related immune cells, and found that a higher cell abundance in the high-risk group (Fig. 8C). The metabolic and immune characteristics were analyzed in GSE14520 (Fig. S3), HCCDC18 (Fig. S4), and GSE76427 (Fig. S5) cohort, and we obtained parallel results as well.

### Verifying the robustness of the risk model at the single cell level

**Figure 9:**
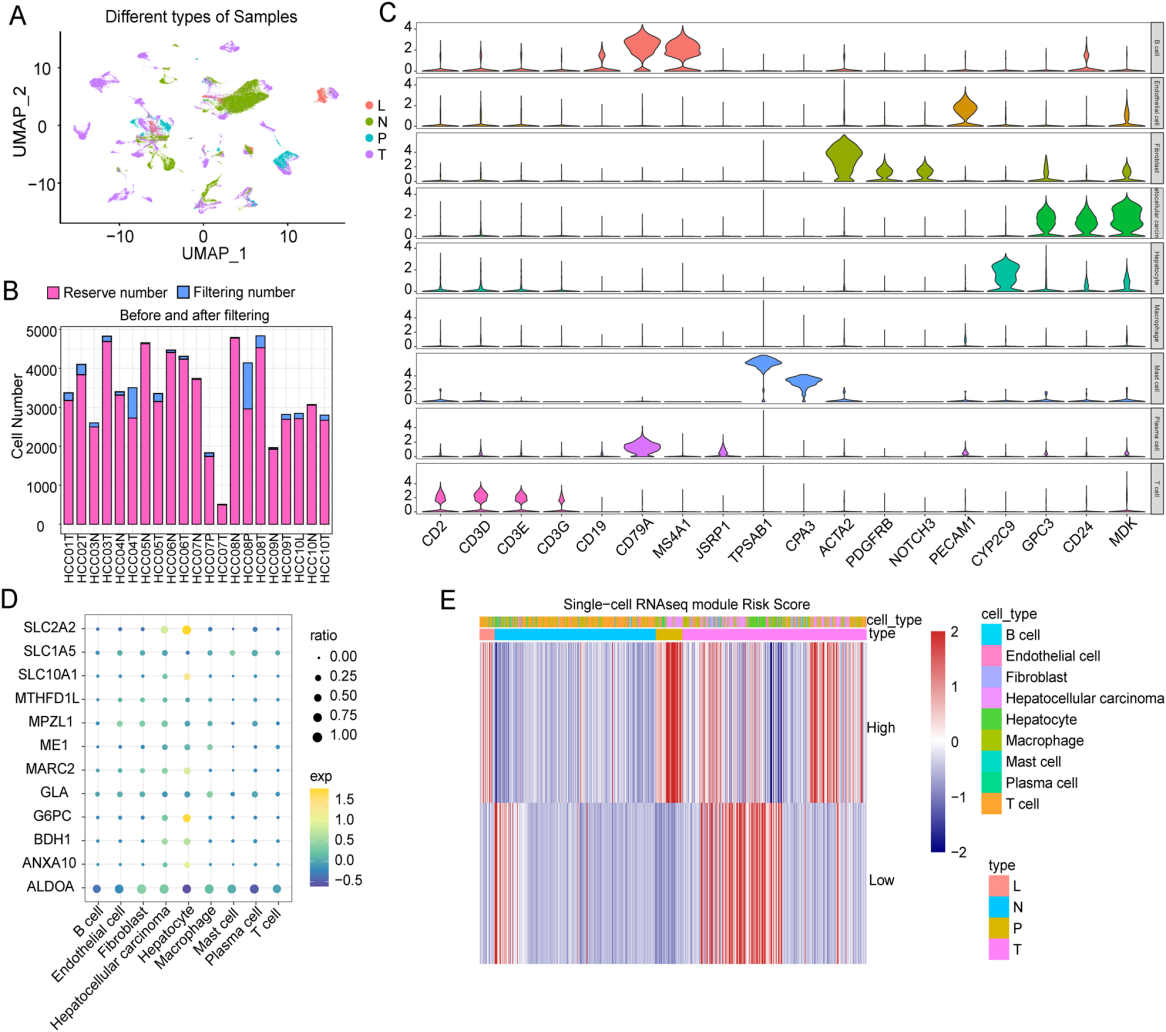
Verifying the accuracy of the risk score at the single cell level. (**A**) The UMAP shows the distribution of different sample types. (**B**) Statistics of cell number before and after filtration. (**C**) The expression of the classical markers in different cell subsets. (**D**) Expression of prognostic genes in different cell subgroups. (**E**) The heatmap of high-risk and low-risk scores under different sample types via ssGSEA.

To further verify the robustness of the risk model, we obtained a single-cell dataset of HCC and conducted cell filtering and clustering using the Seurat package. The uniform manifold approximation and projection (UMAP) dimensionality reduction analysis revealed clear clustering of cells from HCC tissues (T), portal vein tumor thrombus (P), lymph nodes (L), and adjacent nontumor tissue (N) (Fig. 9A). We assessed the cell number in each sample before and after filtration (Fig. 9B) and grouped the cells using FindClusters and annotated them with different markers (Fig. 9C). We then analyzed the expression of genes that were used to construct the risk model across different cell types. We found that *SLC2A2*, *SLC10A1*, and *G6PC* were highly expressed in the hepatocyte but relatively lowly expressed in HCC cells (Fig. 9D). Furthermore, we observed that the risk score was low in normal tissues but high in tumor tissues (Fig. 9E), indicating the robustness of the risk model.

## Discussion

Under aerobic conditions, hepatocytes predominantly metabolize glucose into pyruvate, which is then transported to mitochondria for ATP production through oxidative phosphorylation^21^. However, under hypoxic conditions, such as in tumor microenvironments, the metabolic behavior of hepatocytes shifts towards aerobic glycolysis, leading to the conversion of pyruvate into lactic acid. This metabolic shift is known as the Warburg effect, a hallmark of tumor metabolism^22^. Under sufficient oxygen conditions, tumor cells use glycolysis, providing energy and biosynthetic intermediates for the rapid growth of tumor cells. Several studies have shown that metabolic rewiring in tumor cells can regulate the activity of signaling pathways and modulate gene expression programs that drive cancer invasion and migration^23^. In particular, metabolites such as pyruvate can directly contribute to the invasion and migration of cancer cells^24^. Therefore, identifying genes related to pyruvate metabolism may provide valuable insights into the molecular mechanisms and clinical management of HCC.

In this study, we identified CNV or SNV mutations in several genes related to pyruvate metabolism in liver cancer samples, including *LDHA*, *MPC1*, and *MPC2*. LDHA is one of the key enzymes in glycolysis. Wang and his colleagues discovered that lncRNA HULC binds directly to LDHA and PKM2, affecting their cellular localization, phosphorylation level, and enzyme activity, thereby promoting aerobic glycolysis in hepatoma cells^15^. The mitochondrial pyruvate carriers (MPC1 and MPC2) form hybrid complexes embedded in the mitochondrial inner membrane^25,26^. The loss of MPCs leads to pyruvate transport disorders, which triggers the Warburg effect, while the restoration of MPC function can alter the metabolism and growth characteristics of tumor cells under various conditions^27^. Moreover, recent studies revealed that wild-type p53 induces high expression of the p53-upregulated modulator of apoptosis (PUMA) and is associated with poor prognosis in patients with HCC^9^. Besides, PUMA was found to inhibit pyruvate-driven oxidative phosphorylation and promote glycolysis by interacting with MPCs^9^. However, the combination therapy of miriplatin-TACE and radiotherapy was reported to produce synergistic anti-tumor effects on hepatoma cells through PUMA-mediated apoptosis and cell cycle arrest, which may be efficient for regional terminal HCC^28^. A comprehensive understanding of the metabolic pathways and regulatory factors in HCC is crucial for controlling cancer progression.

We divided patients with HCC into three subtypes based on pyruvate metabolism-related genes, which exhibited significantly different survival rates. Our analysis revealed that poor prognosis in the C3 subtype was associated with the enrichment of p53, MAPK, and TGF-β signaling pathways. Extensive studies have shown that p53 can respond to various cellular stress signals and inhibit cancer by inducing cell cycle arrest or apoptosis. For instance, Zhu et al. revealed that p53 deficiency affects cholesterol esterification and aggravates the occurrence of liver cancer^29^. Besides, Qin et al. identified a long noncoding RNA p53-Stabilizing and Activating RNA, which binds to heterogeneous nuclear ribonucleoprotein K and enhances its deSUMOylation, promotes p53 signaling, and inhibits HCC^30^. The abnormal activation of the MAPK signaling pathway is also closely associated with the occurrence of liver cancer. Han et al. demonstrated that the RNA-binding protein PNO1 could promote autophagy and inhibit apoptosis of hepatoma cells through the MAPK signaling pathway^31^. On the other hand, inactivation mutation of p90 ribosomal S6 kinase 2 was found to activate the MAPK signaling pathway and enhance cholesterol biosynthesis, sensitizing HCC cells to sorafenib treatment^32^. Furthermore, abnormal signals in the TGF-β pathway play an essential immunomodulatory part in the HCC microenvironment^33^. Wu et al. found a positive feedback loop mediated by lnc-UTGF in the TGF-β signaling pathway, which promotes HCC metastasis^34^. These findings may provide new therapeutic strategies and targets for liver cancer.

Our study employed six machine learning algorithms to screen and identify 13 key genes significantly associated with the prognosis of HCC. We subsequently constructed a risk model based on these genes, which exhibited excellent predictive efficacy and held promise in guiding the clinical treatment and prognosis evaluation of HCC. Previously, a variety of prognostic models for HCC have been developed. Liang et al. reported that a novel ferroptosis-related gene signature can be used for prognostic prediction in HCC, and the ROC curve showed that the AUC reached 0.800 at 1 year, 0.690 at 2 years, and 0.668 at 3 years, suggesting a good predictive capacity of the signature^35^. Another study constructed an immunogenomic characteristics for molecular classification in HCC and found that immune risk score could distinguish HCC patients with different prognosis (AUC = 0.704)^36^. The risk model in our study also has a pretty good predictive effect and its robustness was validated in different datasets. These studies suggest that the establishment of prognostic model for HCC might contribute to treatment decision making.

In prior studies, the function of partial key genes in HCC has been reported. MTHFD1L, an enzyme in the folate cycle, can be activated by NRF2 and contributes to the production and accumulation of NADPH to combat oxidative stress in cancer cells^37^. Knockdown of MTHFD1L can enhance the sensitivity of HCC cells to sorafenib by increase the level of oxidative stress, indicating that MTHFD1L in the folate cycle is a promising therapeutic target. MPZL1 is a novel identified HCC metastasis-related gene, which can significantly enhance the migration of HCC cells by inducing the phosphorylation and activation of cortactin^38,39^. MARC2, a member of N-reductive enzyme system, is downregulated in HCC and can promote immune escape^40^. A Study has shown that MARC2 regulates the expression of p27 protein and HNF4A through the Hippo signaling pathway, which is an independent risk indicator for poor prognosis of HCC^41^. BDH1 is a key catalytic enzyme for ketone production in HCC, and its expression in HCC tissues is significantly reduced^42^. In vitro experiment showed that ectopic expression of BDH1 can inhibit the proliferation, migration and invasion of HCC cells, suggesting that BDH1 can be used as a potential diagnostic biomarker. Although existing studies have revealed the molecular mechanism of the prognostic genes in HCC, they are still not in-depth, and more studies are needed in the future to explore the mechanism of their effects on HCC progression.

Our analysis revealed that patients with high-risk scores had a higher clinical stage, pathological grade, immune infiltration, and immune cell abundance. The liver is a vital immune organ with many innate immune cells, and the tumor immune microenvironment is vital in HCC progression, immune tolerance, and immune escape^43^. Studies have revealed that increased T, NK, and NKT cell infiltration in HCC is associated with a better prognosis. In contrast, increased infiltration of regulatory T cells and tumor-associated macrophages are negative prognostic factors^44–47^. Yutaka et al. categorized HCC immune microenvironment into three subtypes: immune high, mid, and low, which have different histopathological features and prognostic impact^48^. Moreover, a recent study identified the spatial heterogeneity of HCC and revealed that CCL15 is enriched in the core region of the tumor site and promotes the immunosuppressive microenvironment by recruiting and polarizing M2-like macrophages, which represented a poor prognosis of patients with HCC^49^. Further studies on the immune microenvironment of HCC will contribute to the development of biomarkers and help predict the efficacy of immunosuppressive therapy.

Although our study provides a potential new strategy for predicting the prognosis of HCC, many limitations remain to be addressed. Our analysis is based on public databases and, therefore, requires further validation using clinical cohorts. Additionally, we did not investigate the protein expression level of the 13 prognostic genes through western blot or immunohistochemistry, nor have we explored the functions of these genes in-depth at the cellular and animal levels. In the future, we intend to further optimize the stability and reliability of our risk model through experimental validation and clinical sample analysis to develop new and effective strategies for the prognosis evaluation of HCC.

## Conclusion

In summary, our study developed a risk model for HCC based on pyruvate metabolism-related genes, which exhibited good prognostic efficacy. Our findings have significant implications for identifying potential prognostic targets and provide a novel strategy for the clinical management of HCC. Further research is needed to refine and optimize the risk model to enhance its stability and generalizability.

## Methods

### Cell culture

SK-HEP-1 is a malignant human liver cancer cell line with strong ability of proliferation, invasion and metastasis^50,51^. The cell line SK-HEP-1 and normal hepatocyte line LO2 were selected for experiment, which were obtained from the Chinese Academy of Sciences (Shanghai, China). Cells were maintained in Dulbecco’s Modified Eagle Medium (Gibco, USA) supplemented with 10% fetal bovine serum (Gibco, USA) and 1% penicillin–streptomycin (Sigma-Aldrich, USA) and cultured in a 37 °C incubator containing 5% CO2. Cells were confirmed to be pollution-free before the experiment.

### Quantitative reverse transcription-PCR

Total RNA was isolated from the cells using an RNeasy Mini Kit (QIAGEN, USA). Then, cDNA was reverse-transcribed by PrimeScript RT Master Kit (Takara, Japan) using the extracted mRNA as the template. All procedures were performed on ice to prevent RNA degradation. A reaction system containing 1 μL cDNA, 10 μL TB Green Premix Ex Taq™ (Takara, Japan), 0.5 μL forward primer and 0.5 μL reverse primer (Sangon Biotech, China), and 8 μL ddH2O was used for amplification in a PCR analyzer (Applied Biosystems, USA). The relative quantification of the target genes was determined using the 2^−ΔΔCt^ method, with GAPDH as the internal reference. The primer sequences are detailed in Table S1.

### Data acquisition

The RNA sequencing data and clinical information of patients with HCC was acquired from TCGA database, comprising 365 tumor samples and 50 paracancerous samples. The raw read count of each sample was normalized by Transcripts Per Million (TPM) method to obtain the relative expression value of mRNA. Two datasets, GSE14520 and GSE76427, were downloaded from the Gene Expression Omnibus (GEO) database, containing 242 and 115 tumor samples, respectively, to serve as validation cohorts^52^. In addition, the expression profile data of the HCCDB18 dataset was obtained from the HCCDB database, and 212 HCC samples were included after screening^53^. The GEO database, GSE149614, was used to obtain single-cell sequencing data for 21 samples. Patients without complete survival status and follow-up information were excluded from subsequent analysis. Moreover, 40 pyruvate metabolism-related genes involved in the “KEGG_PYRUVATE_METABOLISM” pathway were obtained from the Molecular Signatures Database (MSigDB)^54^ are listed in Table S2.

### Construction of molecular subtypes

A consensus cluster analysis of pyruvate metabolism-related genes was performed in TCGA-LIHC using the ConsensusClusterPlus package to determine their molecular subtypes. A chi-square test was adopted to analyze the different clinical features among the three subtypes. Further, the gisticOncoPlot function was utilized to explore the mutation data of the three subtypes based on the TCGA database.

### Functional enrichment analysis

The hallmark gene sets, and KEGG pathway features were obtained from MSigDB^55^. The Gene Set Enrichment Analysis (GSEA) method enriched the hallmark gene sets, while the KEGG pathway features were analyzed using the GSVA package of the R software^56,57^. The enrichment score of patients on each pathway in the TCGA training cohort was calculated, and the pathway with statistical significance between the three subtypes was screened by the Kruskal test method.

### Immune infiltration analysis

ESTIMATE was adopted to assess the differences in immune cell infiltration levels in HCC tissues, including ImmuneScore, StromalScore, and ESTIMATEScore^58^. CIBERSORT, which contains 22 functionally defined human immune subgroups, was used to determine the difference in the proportions of 22 infiltrating human immune cells among the three subtypes^36,59,60^. Additionally, ssGSEA method was employed to determine 28 immune cell scores and examine related pathways, as previously described^61^.

### Establishment of clinical RiskScore model

The limma package was employed to analyze the differential expression between the three subtypes to further filter out the key genes. The threshold for filtering DEGs was set to | log_2_ (Fold Change) |> 1 and false discovery rate < 0.05. Then, univariate Cox analysis of DEGs was performed using the survival package, and cherry-picked genes related to prognosis with a significance level of *P* < 0.001.

The following algorithm was used to evaluate the RiskScore based on the hazard ratio (HR) through multivariate Cox analysis: RiskScore = ΣExp_i_ × HR_i_, where i represents prognostic genes, and Exp represents the mRNA expression level. Finally, the TimeROC package was used to analyze the ROC curve. Spearman's rank correlation test was used to assess associations between RiskScore and clinical features, KEGG pathway, and immune infiltration.

### Single-cell sequencing data

The single-cell data was analyzed using the Seurat R package. The data from 21 samples were log-normalized for data standardization. Highly variable genes were screened using the FindVariableFeatures function and scaled using the ScaleData function^62^. A UMAP package was used for the dimension reduction analysis of cells (dim = 40), and the cells were clustered using FindNeighbors and FindClusters functions (Resolution = 0.1).

### Statistical analysis

All statistical analyses were performed using GraphPad Prism (version 9.3.0) and R software (version 3.6.0) were used to perform statistical analyses. Univariate and multivariate Cox analyses were performed to identify significant prognostic genes. Kaplan–Meier survival analysis and log-rank test were applied to establish the survival curves and compare the differences. Two-tailed *P*-values < 0.05 were considered statistically significant.

## Supplementary Information

Supplementary Information.

## Data Availability

All data involved in this study are available from corresponding author upon rational requirement. The RNA sequencing data were obtained from the TCGA database (https://www.cancer.gov/tcga), GEO database (GSE14520 and GSE76427, https://www.ncbi.nlm.nih.gov/geo/), and HCCDB database (HCCDB18, http://lifeome.net/database/hccdb/). The single-cell sequencing data were obtained from GEO database (GSE149614).
