# Supplementary material for: A novel prognostic model for hepatocellular carcinoma based on pyruvate metabolism-related genes
Source: Sci Rep. 2023 Jun 16;13:9780. doi: 10.1038/s41598-023-37000-8 (PMC10275940; doi:10.1038/s41598-023-37000-8)
Supplement: Supplementary file 1 — Supplementary Information. [file 41598_2023_37000_MOESM1_ESM.pdf]

Supplementary information

A novel prognostic model for hepatocellular carcinoma based on pyruvate metabolism-related genes

Qingmiao Shi[1#], Chen Xue[1#], Yifan Zeng[1], Xinyu Gu[1], Jinzhi Wang[1], Lanjuan Li[1]*

[1]State Key Laboratory for Diagnosis and Treatment of Infectious Diseases, National Clinical Research Center for Infectious Diseases, National Medical Center for Infectious Diseases, Collaborative Innovation Center for Diagnosis and Treatment of Infectious Diseases, The First Affiliated Hospital, Zhejiang University School of Medicine, Hangzhou, 310003, China.

[#]These authors contributed equally.

***Corresponding author: Lanjuan Li**

State Key Laboratory for the Diagnosis and Treatment of Infectious Diseases, The First Affiliated Hospital, Zhejiang University School of Medicine, 79 Qingchun Rd., Hangzhou City 310003, China. Tel: 86-571-87236458; Fax: 86-571-87236459

E-mail: ljli@zju.edu.cn

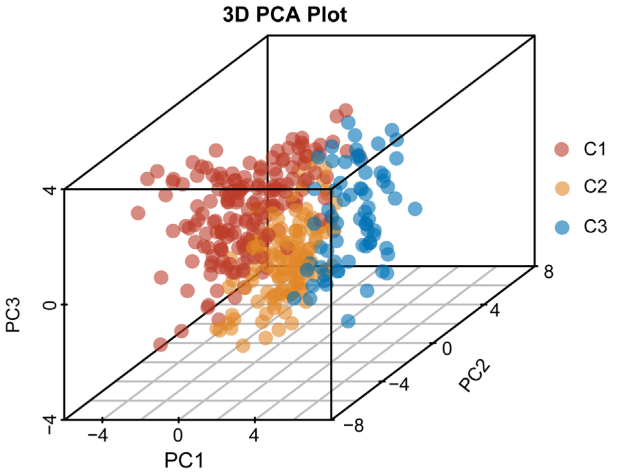

Figure S1. Three-dimensional principal component analysis suggesting a conspicuous discrimination between the three molecular subtypes.

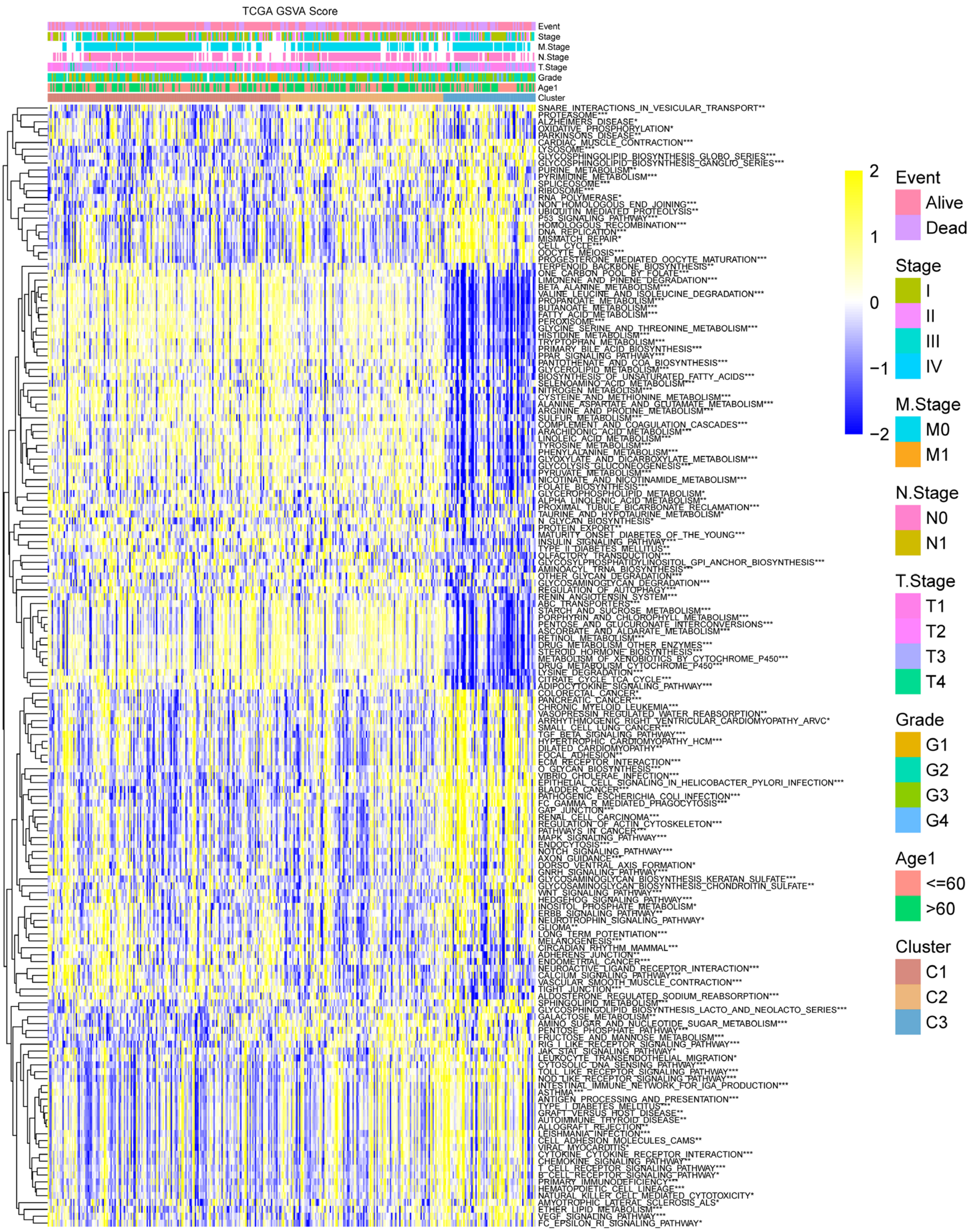

Figure S2. Heatmap of pathway enrichment analysis of the three clusters in the TCGA dataset.

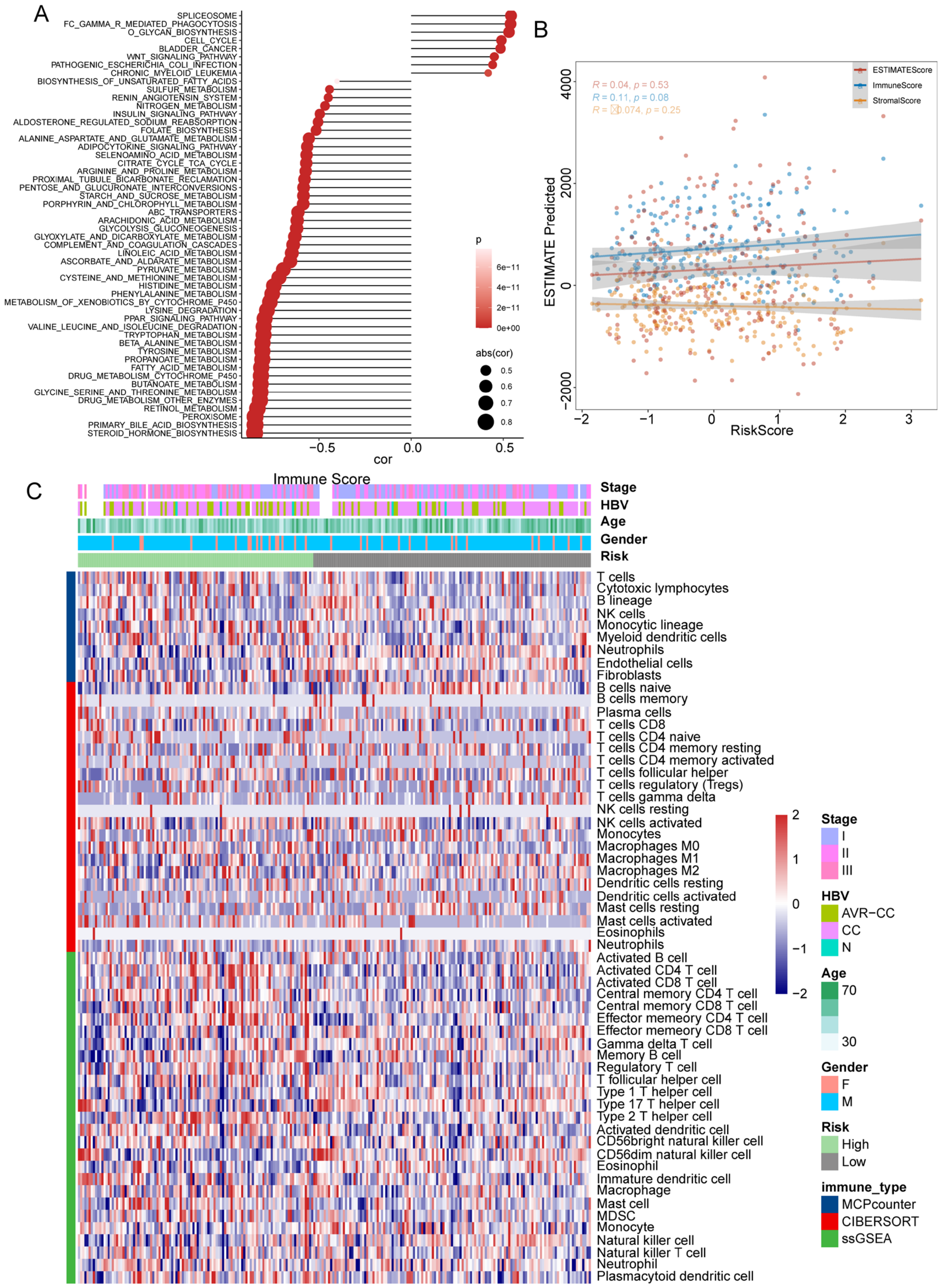

Figure S3. Body characteristics under different risk states in the GSE14520 database. (A) Correlation analysis between pathway score and risk score. (B) Correlation analysis between risk score and ESTIMATE predicted immune score. (C) Heatmap of immune cell abundance evaluated by various algorithms in the high-risk and low-risk groups.

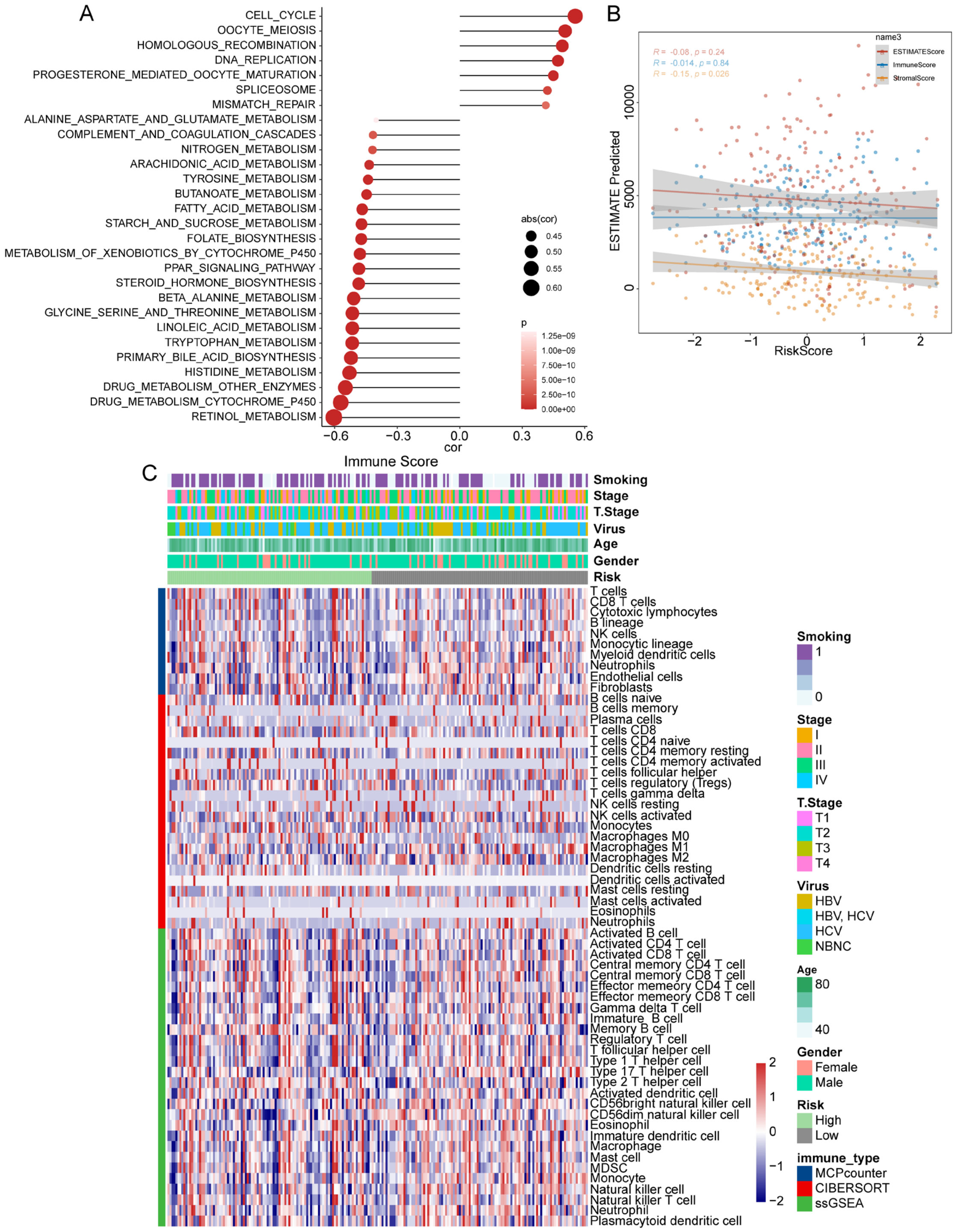

Figure S4. Body characteristics under different risk states in the HCCDC18 database. (A) Correlation analysis between pathway score and risk score. (B) Correlation analysis between risk score and ESTIMATE predicted immune score. (C) Heatmap of immune cell abundance evaluated by various algorithms in the high-risk and low-risk groups.

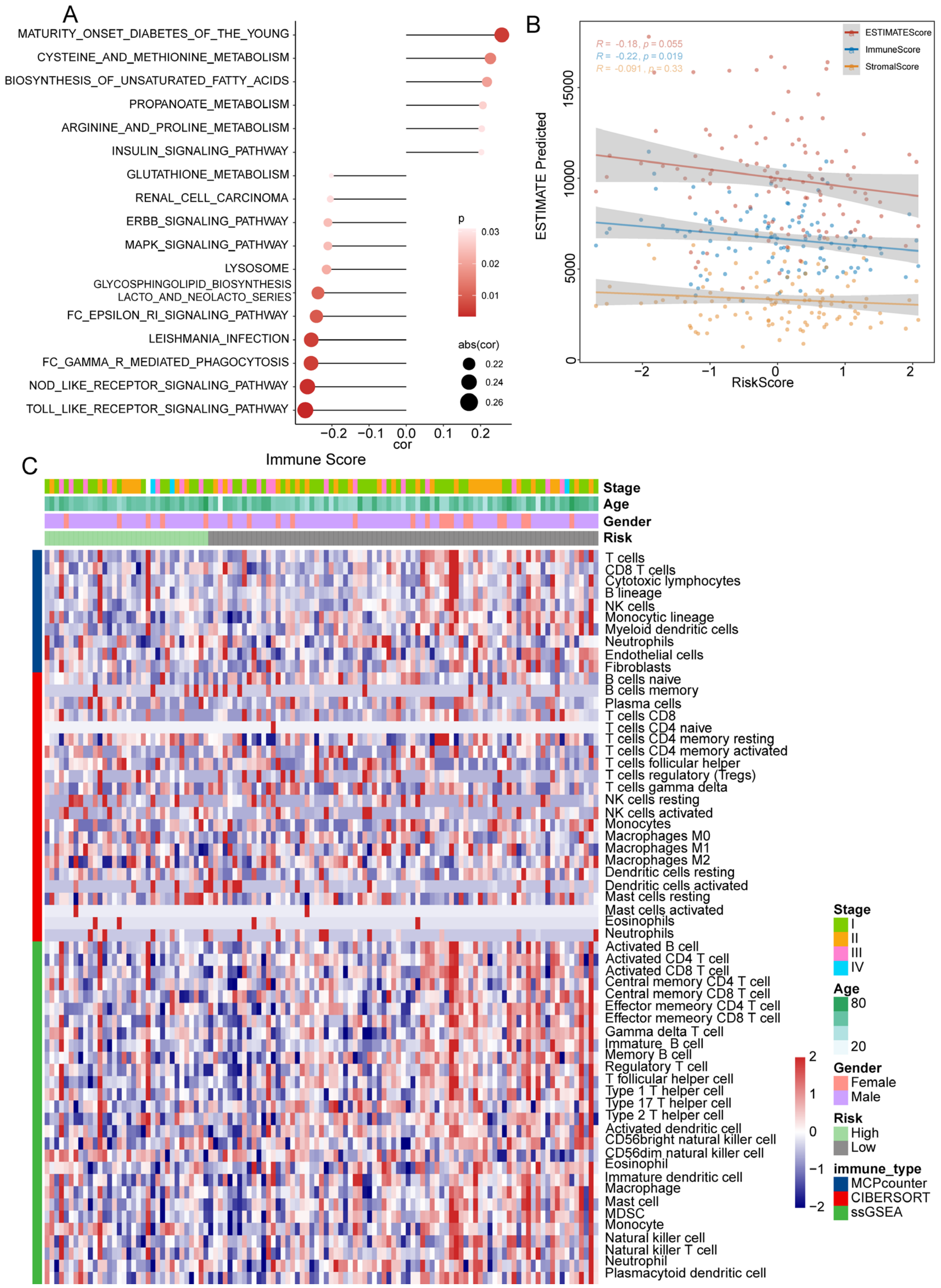

Figure S5. Body characteristics under different risk states in the GSE76427 database. (A) Correlation analysis between pathway score and risk score. (B) Correlation analysis between risk score and ESTIMATE predicted immune score. (C) Heatmap of immune cell abundance evaluated by various algorithms in the high-risk and low-risk groups.

Table S1. The information of primers sequences for qRT-PCR.

Primer name	Sequence (5'-3')	Base
SLC2A2-F	CATCACTGCTGTGCTGGGTT	20
SLC2A2-R	TTGGGTTCATTGAGTATGAGATTG	24
SLC1A5-F	TTTTGGCGGCTGTAGTTGTGGG	22
SLC1A5-R	GGGAGTGTTTCTGTTATTGTGGAGGGA	27
SLC10A1-F	TGGGAAATGGCACCTACAAA	20
SLC10A1-R	TACTGGAAATGCTGGAGAAAGA	22
MTHFD1L-F	CGTTTGGAGTGAAAGGAGGAGC	22
MTHFD1L-R	CAAGTTATTGGCAGCGGTGAT	21
MPZL1-F	GCACAAGGTGGTAAGATAAAGG	22
MPZL1-R	GGTGACCAAACCTGATCCCT	20
ME1-F	ATAAACACTTGGAAGAGGGTCG	22
ME1-R	ACATCTGGGAGCGGACAAA	19
MARC2-F	CGGATGGTGTAGTGATGAGTGA	22
MARC2-R	TCCAGGGTAAAGATAGTTGAAGG	23
JPT1-F	TTTCTAGTAGTAGATTGGAGGGAAAGC	27
JPT1-R	GGTGCCTGTGGACTGTTTATGG	22
GLA-F	GACCTCGCTCTTATACCATCGC	22
GLA-R	CTTGAAGTCCATTCATAGAACCCTA	25
G6PC1-F	ACCACCAAGCCTGGAATAACT	21
G6PC1-R	ATCACGGACACCAAGATGAAC	21
BDH1-F	GGCCATTCTAACACCCGTTGC	21
BDH1-R	AATAGTGGGCGTCTTGCTCCA	21
ANXA10-F	GTACCCACCACCACTGTATGA	21
ANXA10-R	AATGTCCTCTTGGAGGTTATTG	22
ALDOA-F	TGACCACCACATCTACCTGGAAGGC	25
ALDOA-R	CAGGAAGGTGATCCCAGTGACAGC	24

Table S2. The gene set of 40 pyruvate metabolism-related genes

No.	Gene	No.	Gene
1	ACACA	21	HAGHL
2	ACACB	22	LDHA
3	ACAT1	23	LDHAL6A
4	ACAT2	24	LDHAL6B
5	ACOT12	25	LDHB
6	ACSS1	26	LDHC
7	ACSS2	27	LDHD
8	ACYP1	28	MDH1
9	ACYP2	29	MDH2
10	AKR1B1	30	ME1
11	ALDH1B1	31	ME2
12	ALDH2	32	ME3
13	ALDH3A2	33	PC
14	ALDH7A1	34	PCK1
15	ALDH9A1	35	PCK2
16	DLAT	36	PDHA1
17	DLD	37	PDHA2
18	GLO1	38	PDHB
19	GRHPR	39	PKLR
20	HAGH	40	PKM

Note: The gene set was downloaded from the following site: http://www.gsea-msigdb.org/gsea/msigdb/human/geneset/KEGG_PYRUVATE_METABOLISM.html?keywords=Arginine%20metabolism
